# Prediction of clinically significant prostate cancer using radiomics models in real-world clinical practice: a retrospective multicenter study

**DOI:** 10.1186/s13244-024-01631-w

**Published:** 2024-02-29

**Authors:** Jie Bao, Xiaomeng Qiao, Yang Song, Yueting Su, Libiao Ji, Junkang Shen, Guang Yang, Hailin Shen, Ximing Wang, Chunhong Hu

**Affiliations:** 1 Department of Radiology, The First Affiliated Hospital of Soochow University, 188#, Shizi Road, Suzhou, Jiangsu 215006 China; 2 Scientific Marketing, Siemens Healthineers, Shanghai 430#, Linqing Road, Shanghai, 201318 China; 3 Department of Radiology, The People’s Hospital of Taizhou, 210#, Yingchun Road, Taizhou, Jiangsu 225399 China; 4 Department of Radiology, Changshu NO.1 People’s Hospital, 1#, Shuyuan Street, Changshu, Jiangsu 215501 China; 5 Department of Radiology, The Second Affiliated Hospital of Soochow University, 1055#, Sanxiang Road, Suzhou, Jiangsu 215004 China; 6 Shanghai Key Laboratory of Magnetic Resonance, East China Normal University, 3663#, Zhongshanbei Road, Shanghai, China; 7 Department of Radiology, Suzhou Kowloon Hospital, Shanghai Jiaotong University School of Medicine, 118#, Wanshen Street, Suzhou, Jiangsu 215028 China

**Keywords:** Neoplasms, Prostatic, Magnetic resonance imaging, Random forest, Retrospective study

## Abstract

**Purpose:**

To develop and evaluate machine learning models based on MRI to predict clinically significant prostate cancer (csPCa) and International Society of Urological Pathology (ISUP) grade group as well as explore the potential value of radiomics models for improving the performance of radiologists for Prostate Imaging Reporting and Data System (PI-RADS) assessment.

**Material and methods:**

A total of 1616 patients from 4 tertiary care medical centers were retrospectively enrolled. PI-RADS assessments were performed by junior, senior, and expert-level radiologists. The radiomics models for predicting csPCa were built using 4 machine-learning algorithms. The PI-RADS were adjusted by the radiomics model. The relationship between the Rad-score and ISUP was evaluated by Spearman analysis.

**Results:**

The radiomics models made using the random forest algorithm yielded areas under the receiver operating characteristic curves (AUCs) of 0.874, 0.876, and 0.893 in an internal testing cohort and external testing cohorts, respectively. The AUC of the adjusted_PI-RADS was improved, and the specificity was improved at a slight sacrifice of sensitivity. The participant-level correlation showed that the Rad-score was positively correlated with ISUP in all testing cohorts (*r* > 0.600 and *p* < 0.0001).

**Conclusions:**

This radiomics model resulted as a powerful, non-invasive auxiliary tool for accurately predicting prostate cancer aggressiveness. The radiomics model could reduce unnecessary biopsies and help improve the diagnostic performance of radiologists’ PI-RADS. Yet, prospective studies are still needed to validate the radiomics models further.

**Critical relevance statement:**

The radiomics model with MRI may help to accurately screen out clinically significant prostate cancer, thereby assisting physicians in making individualized treatment plans.

**Key points:**

• The diagnostic performance of the radiomics model using the Random Forest algorithm is comparable to the Prostate Imaging Reporting and Data System (PI-RADS) obtained by radiologists.

• The performance of the adjusted Prostate Imaging Reporting and Data System (PI-RADS) was improved, which implied that the radiomics model could be a potential radiological assessment tool.

• The radiomics model lowered the percentage of equivocal cases. Moreover, the Rad-scores can be used to characterize prostate cancer aggressiveness.

**Graphical Abstract:**

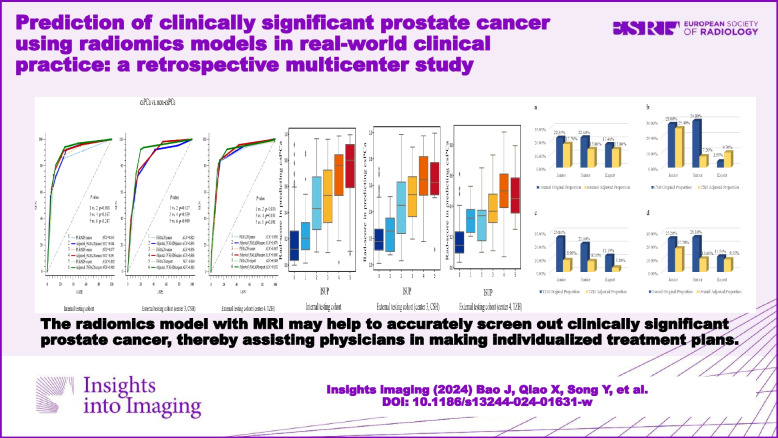

**Supplementary Information:**

The online version contains supplementary material available at 10.1186/s13244-024-01631-w.

## Background

Prostate cancer (PCa) is among the most common cancers affecting the male population, whose incidence has been increasing every year [1]. The International Society of Urological Pathology (ISUP) grade groups is currently considered the best prognostic factor for determining PCa aggressiveness and therapeutic schedule [2]. Digital rectal examination and prostate-specific antigen (PSA) tests, followed by transrectal ultrasound (TRUS) guided biopsy, are widely used diagnostic approaches for PCa; yet, these methods have been associated with an elevated rate of overdiagnosis or under-diagnosis [3].

Multiparametric magnetic resonance imaging (mpMRI), including T2 weighted imaging (T2WI), diffusion-weighted imaging (DWI), and apparent diffusion coefficient (ADC) maps derived from DWI and dynamic contrast-enhanced (DCE), are being increasingly used for the detection of PCa [4, 5]. Prostate Imaging Reporting and Data System (PI-RADS) has been designed to standardize image acquisition techniques and interpretation of prostate MRI [6]; however, despite the widespread application in clinical practice, PI-RADS is a semi-quantitative assessment affected by the subjectivity and variability of a radiologist, with only moderate to good interobserver agreement [7]. A five-point scale PI-RADS lacks objective distinction between inflammatory and tumor lesions. It is also not able to assess the aggressiveness of prostate cancer. Subsequently, numerous studies have validated PI-RADS but have also shown some limitations, such as several specific assessment criteria requiring clarification or adjustment [6, 8]. The vocabulary and subjective assessments of different radiologists are of significant importance for the validity of the report. While lesions scored 1 or 2 indicate that clinically significant cancer is unlikely and lesions scored 4 or 5 indicate that clinically significant cancer is likely present, lesions scored 3 are intermediate or equivocal lesions that pose a significant challenge to clinical management [6, 9].

With the rapid development of artificial intelligence, the radiomics features include high-dim features and some identifiable by the naked eye [10]. Radiomics is a non-invasive quantitative method used to evaluate tumor heterogeneity and complexity [11]. Previous studies have applied radiomics to stratify risk categories of histological Gleason grade and predict extracapsular extension, lymph node metastasis, and recurrence-free survival in the prostate [12–15]. The potential value of radiomics for diagnosing and predicting PCa using MRI has also been reported. For example, recent single-center studies [16, 17] have employed radiomics analysis of MRI for the detection of clinically significant prostate cancer (csPCa); however, the high variation of pathological characteristics of PCa and the imbalance in single-center data can easily lead to overfitting, hindering the generalization of the radiomics model. In particular, identifying the obstacles to predicting csPCa would more effectively overcome the lack of a universally validated radiomic tool and the endpoint of screening out csPCa. Indeed, it is necessary to develop an alternative and robust tool using multi-center data to quantify the accuracy and generalizability of the new tool in assessing PCa characteristics more effectively.

This study aimed to develop and validate robust and generalizable machine learning models using multicenter data for the diagnosis of csPCa and evaluate the auxiliary diagnostic role in improving the diagnostic performance of different radiologists, attempting to expand the potential value in decreasing unnecessary biopsies for specific PI-RADS category 3 patients. Moreover, we further evaluated the correlation with radiomics scores and the histopathologic ISUP grade groups to assess the ability of pathological characteristics using multicenter MRI data to evaluate PCa aggressiveness.

## Materials and methods

A total of 1616 patients with biopsy-proven PCa were reviewed from databases of 4 collaborating centers (i.e., center 1, center 2, center 3, and center 4) between January 2015 and December 2021. Inclusion criteria of this study were as follows: (1) biopsy-naive men who underwent standard prostate 3.0-T MRI within 4 weeks before biopsy and (2) biopsy-naive men who underwent standard transrectal ultrasonography (TRUS)/MRI fusion or cognitive fusion targeted biopsy and systematic biopsy. Exclusion criteria were as follows: (1) absence of prostate biopsy or radical prostatectomy (RP) results; (2) incomplete MRI sequence or poor image quality (displacement, gas, or motion artifacts) that cannot be used for diagnosis; (3) previous history of biopsy or surgery or treatment for PCa.

**Fig. 1:**
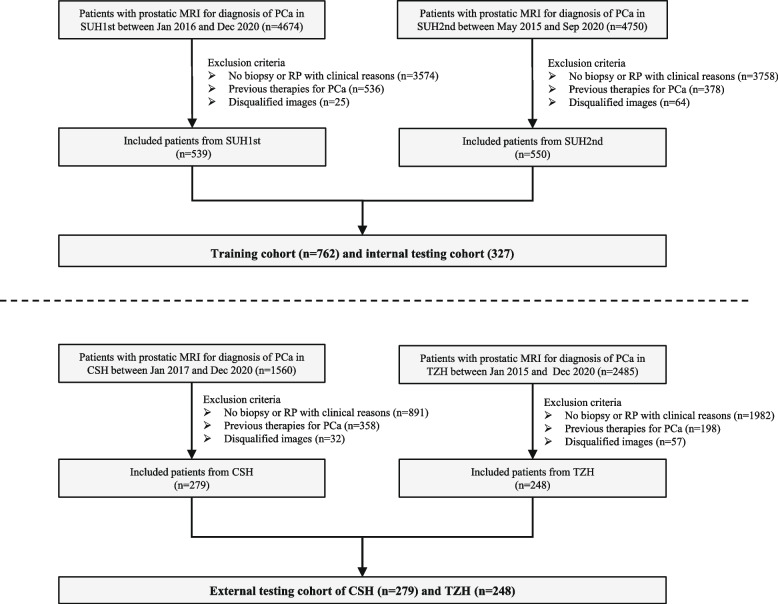
The study flow chart. Notes: Center 1, SUH1st, the First Affiliated Hospital of Soochow University; center 2, SUH2nd, the Second Affiliated Hospital of Soochow University; center 3, CSH, Changshu NO.1 People’s Hospital; center 4, TZH, People’s Hospital of Taizhou; PCa, prostate cancer

Finally, 539 patients from center 1, 550 from center 2, 279 from center 3, and 248 from center 4 were included. The final cohort comprised 1616 patients with clinical indications of prostate MRI; the study flow diagram is shown in Fig. 1.

### MRI acquisition and PI-RADS assessment

All mpMRI exams were performed using 3.0-T MRI scanners with pelvic phased array coils. The mpMRI included T2WI in three planes: DWI, ADC maps in the axial plane, and DCE. The details of the institutional mpMRI protocols are shown in Table S1.

This multi-center study set up a PI-RADS assessment team to score the enrolled patients. PI-RADS assessment was divided into three steps: first, according to PI-RADS version 2.1 [6], the PI-RADS were assessed by two radiologists from center 1 and center 3 (reader 1 and reader 2 with 3 and 6 years of experience in prostate imaging, respectively) evaluating index lesions based on T2WI, DWI/ADC, and DCE imaging, namely PI-RADS_junior_. The other steps of the assessment are described in Supplement Section 1.

The entire three-dimensional volume of interest (VOI) of the lesion was segmented on consecutive T2WI axial slices using ITK-SNAP (open-source software, v3.8.0; www.itksnap.org) based on histopathologic-imaging matching. The details of the manual segmentations are summarized in Supplement Section 2.

### Histopathology

As a standard part of patient management, patients who scored PI-RADS ≥ 3 underwent targeted standard transrectal ultrasonography (TRUS)/MRI fusion or cognitive fusion targeted biopsy in conjunction with systematic biopsy. Uropathologists reviewed the histopathological slides using the 2014 ISUP standard [18]. The ISUP ≤ 2 group was designated as the non-csPCa group, with ISUP > 2 as the csPCa group; the remaining details of the histopathology findings are summarized in Supplement Section 3.

### Radiomics feature extraction

Radiomics features were extracted with FeatureExplorer (v0.5.2) [19], open-source software for radiomics study based on PyRadiomics (v3.0). The details of this procedure were declared in Supplement Section 4. Finally, a total of 292 features were extracted from three sequences. ComBat was used to alleviate the differences in feature distributions among different centers [20].

### Feature selection and radiomics model development

We randomly split the data of center 1 and center 2 at the patient level into a training cohort (*n* = 762) and an internal testing (*n* = 327) cohort in a 7:3 ratio. The data from center 3 (*n* = 279) and center 4 (*n* = 248) were used as two separate external testing cohorts.

In order to remove the imbalance from the training data set, we performed up-sampling by repeating random cases to equal the number of positive/negative samples. The *z*-score was used to normalize each feature by subtracting the mean value and dividing it by the standard deviation. The dimension reduction was applied to the normalized feature. Pearson correlation coefficient (PCC) was calculated for each pair of two features, one of which was dropped if the PCC value was > 0.99. Analysis of variance (ANOVA) was used for feature selection, and the *F*-value of each feature was calculated based on the labels in the training cohort. The selected features for predicting csPCa are summarized in Table S2. Finally, the random forest (RF), support vector machine (SVM), logistic regression (LR), and linear discriminant analysis (LDA) models were trained on the selected features to build the radiomics model separately. We used 5-fold cross-validation on the training cohort to determine the hyper-parameters of the pipeline, including the number of selected features, the kernel, or the regularization parameter of the four classifications, after which the hyper-parameters that achieved the highest cross-validation performance were used to train the final model on the whole training cohort. The details of the pipeline of the machine models are shown in Figure S1. The prediction of the final model was used as the radiomics score (Rad-score) in the subsequent analysis.

**Fig. 2:**
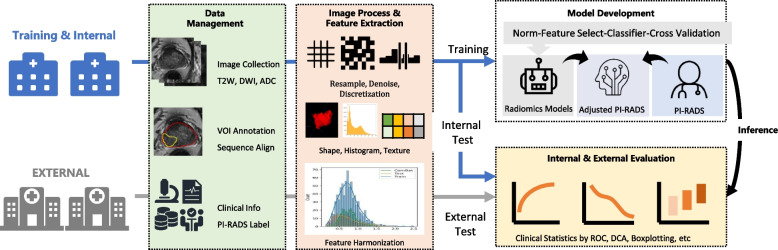
The workflow of the development and testing of the radiomics models. First, the index lesion was manually segmented on axial T2WI for radiomics analysis. Second, radiomics features were extracted from T2WI, DWI, and ADC. Third, the random forest (RF), support vector machine (SVM), logistic regression (LR), and linear discriminant analysis (LDA) were trained on the selected features to build the radiomics model separately, and the corresponding radiomics scores (Rad-score) were acquired by the logistic regression model. Fourth, in the process of testing, the models were tested with an internal testing cohort and two external testing cohorts. ROC, receiver operating characteristics; DCA, decision curve analysis; PI-RADS, Prostate Imaging Reporting and Data System

First, the radiomics models for predicting csPCa were compared with the discrimination performance of PI-RADS_junior_, PI-RADS_senior_, and PI-RADS_expert_ of radiologists. Second, each patient in testing cohorts had a Rad-score; when the Rad-score was higher than the cut-off value, the patient’s assessment was deemed as positive. Conversely, the patient’s assessment was deemed negative when the Rad-score was smaller than the cut-off value. Indeed, the PI-RADS_junior_, PI-RADS_senior_, and PI-RADS_expert_ were upgraded when the radiomics models produced a positive assessment, except for the highest score of 5. Conversely, the three PI-RADS of radiologists were downgraded if the radiomics models produced a negative assessment, except for the lowest score of 1. The three adjusted PI-RADS were denominated as adjusted_PI-RADS_junior_, adjusted_PI-RADS_senior,_ and adjusted_PI-RADS_expert_, respectively. Third, we compared the Rad-score distribution among the sub-groups with different ISUP. The flowchart of the data processing, including data annotation, feature extraction and selection, and model building and comparison, is shown in Fig. 2.

### Statistical analysis

Variables were expressed as median and range, or mean and standard deviation according to the normality test. An independent *t*-test was used to compare the normally distributed continuous variables. The Shapiro–Wilk test was used to test for normality. The Mann–Whitney test was used to compare non-normally distrusted continuous variables.

All models were evaluated by the receiver operating characteristic (ROC) curves. The area under the ROC curve (AUC) was also calculated. The cut-off was determined according to the maximum Youden index on the training cohort, and the corresponding confusion matrix was calculated to estimate the sensitivity, specificity, positive predictive value (PPV), and negative predictive value (NPV). The DeLong test was used to compare the ROC curve of the models. Spearman analysis was used to evaluate the correlation between the Rad-score and ISUP. The summary receiver operating characteristic (SROC) curve has been recommended to represent the performance of a diagnostic test based on data from a meta-analysis; therefore, we used the SROC to evaluate the diagnosis performance. Decision curve analysis (DCA) was used to estimate the risk threshold for the net benefits; a radiomics quality score checklist was used to evaluate the quality of this study (Supplement Section 5).

The statistical analysis was conducted with Python (version 3.8.3), R Studio (version 1.4), and MedCalc software (version 19.6.4). A two-sided *p* < 0.05 was considered statistically significant.

## Results

### Study characteristics

A total of 1616 patients from four centers were enrolled in this study. The selected patients from center 1 and center 2 were merged and then randomly divided into a training cohort (*n* = 762, 243 (31.9%) csPCa, 135 (17.7%) ciPCa, and 384 (50.4%) benign) and an internal test cohort (*n* = 327, 104 (31.8%) csPCa, 58 (17.7%) ciPCa, and 165 (50.5%) benign). The patients from center 3 and center 4 were collected for external test cohorts (center 3: *n* = 279, 65 (23.3%) PCa, 49 (17.6%) ciPCa, and 165 (59.1%) BPH; center 4: *n* = 248, 120 (48.4%) PCa, 24 (9.7%) ciPCa, and 104 (41.9%) BPH). Random re-splitting showed no significant differences between the training cohort and internal test cohort in terms of age, PSA, D_max, position of lesions, seminal vesicle invasion (SVI), extracapsular extension (ECE), and lymph node invasion (LNI) (all *p* > 0.05). The clinical characteristics of the patients from the four centers are shown in Table 1.

**Table 1:** The baseline characteristics of training cohort and internal and external testing cohorts

Variable	Training cohort	Internal testing cohort		External testing cohorts	
	SUH1st (center 1) and SUH2nd (center 2) (*n* = 1089)		*p*	CSH (center 3, *n* = 279)	TZH (center 4, *n* = 248)
No. of subjects	762	327		279	248
Age (years), median (IQR)	69 (64–75)	69 (64–75)	0.828	70 (65–75)	73 (68–78)
PSA level, median (IQR)	11.0 (7.3–21.0)	11.6 (7.2–22.6)	0.344	12.3 (7.5–23.0)	15.5 (6.3–71.9)
0–10 ng/mL, *n* (%)	334 (43.8%)	132 (40.4%)		109 (39.1%)	99 (39.9%)
10–20 ng/mL, *n* (%)	230 (30.2%)	106 (32.4%)		88 (31.5%)	30 (12.1%)
> 20 ng/mL, *n* (%)	198 (25.9%)	89 (27.2%)		82 (29.4%)	119 (47.9%)
D-max (mm), median (IQR)	20.2 (15.5–28.0)	21.4 (15.8–29.4)	0.344	17.0 (12.5–27.3)	40.9 (28.3–52.0)
Prostate zone, *n*			0.719		
PZ, *n* (%)	279 (36.6%)	127 (38.8%)		69 (24.7%)	50 (20.2%)
TZ, *n* (%)	353 (46.3%)	143 (43.7%)		174 (62.4%)	121 (48.8%)
PZ, TZ and AFMS, *n* (%)	130 (17.1%)	57 (17.4%)		36 (12.9%)	77 (31.1%)
MRI index lesion per patient, *n* (%)			0.780		
PI-RADS 1–2	243 (31.9%)	108 (33.0%)		50 (17.9%)	65 (26.2%)
PI-RADS 3	178 (23.4%)	73 (22.3%)		78 (28.0%)	64 (25.8%)
PI-RADS 4	134 (17.6%)	50 (15.3%)		72 (25.8%)	23 (9.3%)
PI-RADS 5	207 (27.2%)	96 (29.4%)		79 (28.3%)	96 (38.7%)
Biopsy ISUP grade, *n* (%)	759	327	0.378	272	246
ISUP 0 (benign)	387 (51.0%)	166 (50.8%)		165 (60.7%)	103 (41.9%)
ISUP 1	70 (9.2%)	32 (9.8%)		7 (2.6%)	8 (3.3%)
ISUP 2	76 (10.0%)	32 (9.8%)		39 (14.3%)	16 (6.5%)
ISUP 3	83 (10.9%)	27 (8.3%)		17 (6.3%)	30 (12.2%)
ISUP 4	66 (8.7%)	24 (7.3%)		33 (12.1%)	46 (18.7%)
ISUP 5	77 (10.1%)	46 (14.1%)		11 (4.0%)	43 (17.5%)
Surgical ISUP grade, *n* (%)	259	117	0.051	7	26
ISUP 1	43 (16.6%)	22 (18.8%)		0 (0.0%)	0 (0.0%)
ISUP 2	55 (21.2%)	27 (23.1%)		3 (42.9%)	6 (23.1%)
ISUP 3	79 (30.5%)	19 (16.2%)		2 (28.6%)	3 (11.5%)
ISUP 4	26 (10.0%)	18 (15.4%)		1 (14.3%)	6 (23.1%)
ISUP 5	56 (21.6%)	31 (26.5%)		1 (14.3%)	11 (42.3%)
Label, *n* (%)	762	327	1.000	279	248
Benign lesion	384 (50.4%)	165 (50.5%)		165 (59.1%)	104 (41.9%)
ciPCa	135 (17.7%)	58 (17.7%)		49 (17.6%)	24 (9.7%)
csPCa	243 (31.9%)	104 (31.8%)		65 (23.3%)	120 (48.4%)
ECE, *n* (%)	239	110	0.925	7	26
Present	90 (37.7%)	42 (38.2%)		1 (14.3%)	12 (46.2%)
Absent	149 (62.3%)	68 (61.8%)		6 (85.7%)	14 (53.9%)
SVI, *n* (%)	255	114	0.828	7	26
Present	38 (14.9%)	16 (14.0%)		0 (0.0%)	5 (19.2%)
Absent	217 (85.1%)	98 (86.0%)		7 (100.0%)	21 (80.8%)
LNI, *n* (%)	123	62	0.695	3	1
Present	8 (6.5%)	5 (8.1%)		0 (0.0%)	0 (0.0%)
Absent	115 (93.5%)	57 (91.9%)		3 (100.0%)	1 (100.0%)

Unless indicated otherwise, data are numbers of patients with percentage in parentheses. *p* value was evaluated by two-tailed *t*-test with unequal variance. Gleason grade (GG) is according to the 2014 International Society of Urological Pathology (ISUP) standards

*Notes: PCa* Prostate cancer, *ciPCa* Clinically insignificant prostate cancer, *csPCa* Clinically significant prostate cancer, *PI-RADS* Prostate Imaging Reporting and Data System, *PZ* Peripheral zone, *TZ* Transition zone, *CZ* Center zone, *AFMS* Anterior fibromuscular stroma, *ECE* Extracapsular extension, *SVI* Seminal vesicle infiltration, *LNI* Lymph node invasion, *D-max* Diameter in greatest dimension

### Diagnosis performance of PI-RADS of three radiologists

The performance of the PI-RADS in predicting csPCa is shown in Figure S2. For csPCa prediction, PI-RADS_expert_ achieved higher AUCs than PI-RADS_junior_ and PI-RADS_senior_ in internal and external testing cohorts (Figure S2). The difference between PI-RADS_junior_ (internal: AUC = 0.845 [0.796–0.894]; center 3: AUC = 0.823 [0.765–0.882]) and PI-RADS_expert_ (internal: 0.892 [0.855–0.929], center 3: 0.884 [0.838–0.930]) in an internal testing cohort (*p* = 0.041) and external testing cohort of center 3 (*p* = 0.003) and the difference between PI-RADS_junior_ (AUC = 0.858 [0.808–0.908]) and PI-RADS_senior_ (AUC = 0.867 [0.818–0.916]) in external testing cohort of center 4 (*p* = 0.046) were statistically significant, while the remaining ones were insignificant (all *p* > 0.05).

### Performance and clinical application of the radiomics model

The performance of the radiomics models using different machine learning algorithms (i.e., RF, SVM, LR, and LDA) is summarized in Figure S3. The cross-validation results in predicting csPCa of four algorithms are summarized in Table S3. The radiomics model using the RF algorithm achieved the highest AUC compared with radiomics models based on the other three algorithms. Indeed, we selected the radiomics model using the RF algorithm in the following application. The radiomics model using the RF algorithm showed the highest predictive performance for csPCa prediction in the internal testing cohort (AUC = 0.874, [0.834–0.915]) (all* p* < 0.05), an external testing cohort of center 3 (AUC = 0.876 [0.831–0.920]) (all* p* < 0.05), and an external testing cohort of center 4 (AUC = 0.893 [0.853–0.933]) (all* p* > 0.05) (Figure S3). The SEN in predicting csPCa was 83.7% (87/104), 87.7% (57/65), and 90.0% (108/120) in the internal testing cohort and external testing cohorts of center 3 and center 4, and the SPE was 78% (174/223), 77.6% (166/214), and 73.2% (94/128), respectively.

**Fig. 3:**
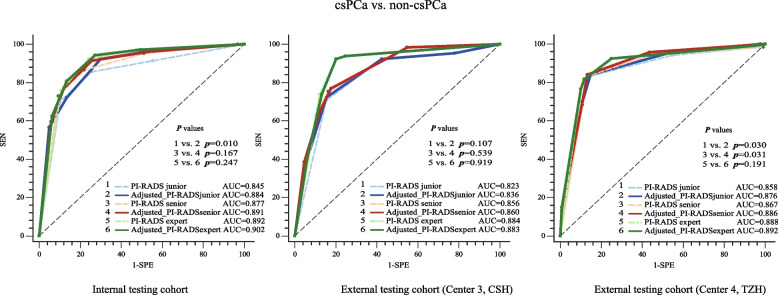
The comparison of diagnosis performance of the adjusted PI-RADS and PI-RADS of three different radiologists in predicting csPCa in an internal testing cohort, an external testing cohort of center 3 and an external testing cohort of center 4. The AUC of adjusted PI-RADS was improved compared with PI-RADS in predicting csPCa; the statistical differences between PI-RADS_junior_ vs. adjusted_PI-RADS_junior_ (*p* = 0.010) in the internal testing cohort, PI-RADS_junior,_ vs. adjusted_PI-RADS_junior_ (*p* = 0.030), and PI-RADS_senior_ vs. adjusted_PI-RADS_senior_ (*p* = 0.031) in external testing cohort of center 4 were significant. Notes: ROC, receiver operating characteristics; AUC, area under ROC curve; center 3, CSH, Changshu NO.1 People’s Hospital; center 4, TZH, People’s Hospital of Taizhou; csPCa, clinically significant prostate cancer

When three PI-RADS of radiologists were adjusted according to the prediction of the radiomics models, their diagnosis performance was improved (Table 2). It is worth mentioning that the SPE of the adjusted PI-RADS of three different level radiologists for csPCa prediction was substantially improved at a slight sacrifice of SEN. As shown in Fig. 3, all performances of three radiologists were improved in predicting csPCa; only the difference of junior radiologist in the internal testing cohort (*p* = 0.01) and the difference of junior and senior radiologists in the external testing cohort of center 4 (*p* = 0.030 and *p* = 0.031) were significant, while the remaining ones in three testing cohorts were insignificant (all *p* > 0.05).

**Table 2:** The diagnosis performance of adjusted PI-RADS in predicting csPCa of three different level radiologists in the internal testing cohort and external testing cohorts of center 3 and center 4

	Internal testing cohort					External testing cohort (center 3, CSH)					External testing cohort (center 4, TZH)				
Adjusted_PI-RADS_junior_	0.884 (0.845–0.923)	92.3 (96/104)	70.0 (156/223)	58.9 (96/163)	95.1 (156/164)	0.836 (0.778–0.893)	92.3 (60/65)	57.5 (123/214)	39.7 (60/151)	96.1 (123/128)	0.876 (0.831–0.921)	84.2 (101/120)	85.2 (109/128)	84.2 (101/120)	85.2 (109/128)
Adjusted_PI-RADS_senior_	0.891 (0.853–0.930)	91.3 (95/104)	74.4 (166/223)	62.5 (95/152)	94.9 (166/175)	0.860 (0.812–0.908)	76.9 (50/65)	82.2 (176/214)	56.8 (50/88)	92.1 (176/191)	0.886 (0.843–0.929)	84.2 (101/120)	86.7 (111/128)	85.6 (101/118)	85.4 (111/130)
Adjusted_PI-RADS_expert_	0.902 (0.867–0.938)	94.2 (98/104)	72.6 (162/223)	61.6 (98/159)	96.4 (162/168)	0.883 (0.839–0.927)	92.3 (60/65)	79.9 (171/214)	58.2 (60/103)	97.2 (171/176)	0.892 (0.849–0.935)	81.7 (98/120)	88.3 (113/128)	86.7 (98/113)	83.7 (113/135)

*Notes: ROC* Receiver operating characteristics, *AUC* Area under ROC curve, *ACC* Accuracy, *SEN* Sensitivity, *SPE* Specificity, *PPV* Positive predictive value, *NPV* Negative predictive value, *center 3, CSH* Changshu NO.1 People’s Hospital, *center 4, TZH* People’s Hospital of Taizhou, *csPCa* Clinically significant prostate cancer, *PI-RADS* Prostate Imaging Reporting and Data System

**Fig. 4:**
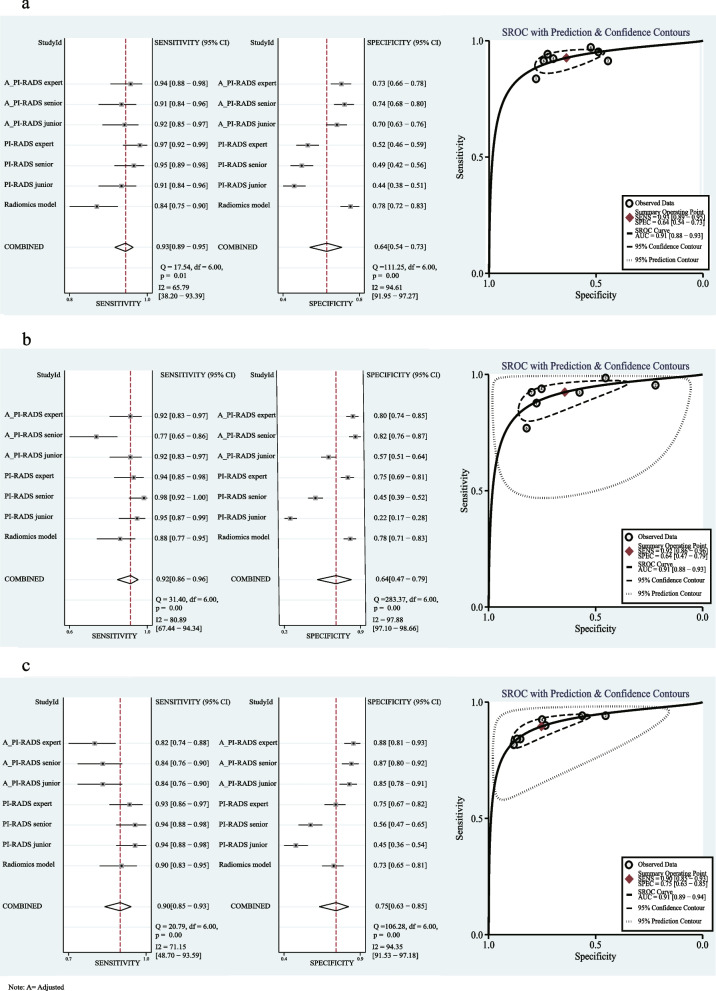
The sensitivity, specificity, and summary receiver operating characteristic (SROC) curves of the radiomics model, PI-RADS of three radiologists, and adjusted PI-RADS of three radiologists in predicting csPCa in the internal testing cohort (**a**), external testing cohort of center 3 (**b**), and external testing cohort of center 4 (**c**). The plots show individual and combined sensitivity, specificity, and area under SROC curves of the different diagnostic methods using meta-regression analysis. Notes: ROC, receiver operating characteristics; AUC, area under ROC curve; SROC, summary receiver operating characteristic; SEN, sensitivity; SPE, specificity; center 3, CSH, Changshu NO.1 People’s Hospital; center 4, TZH, People’s Hospital of Taizhou; PI-RADS, Prostate Imaging Reporting and Data System

**Fig. 5:**
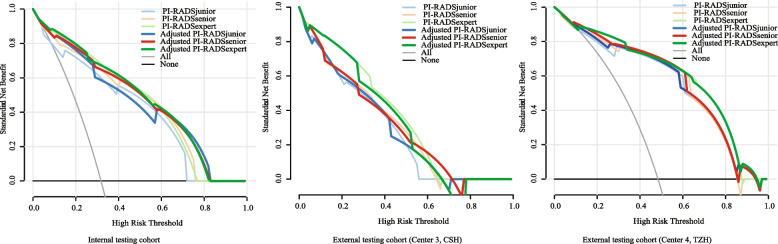
Decision curve analysis (DCA) of clinical usefulness assessment of radiomics model, PI-RADS of three different level radiologists, and adjusted PI-RADS of three different level radiologists in predicting csPCa in the internal testing cohort and external testing cohorts of center 3 and center 4. Notes: center 3, CSH, Changshu NO.1 People’s Hospital; center 4, TZH, People’s Hospital of Taizhou; PI-RADS, Prostate Imaging Reporting and Data System

To provide a comprehensive explanation, the independent and integrated effects of the PI-RADS of three different level radiologists, radiomics model, and adjusted PI-RADS of three different level radiologists were evaluated in the internal testing cohort and an external testing cohorts of center 3 and center 4 using SROC curves and forest plots with a Bayesian meta-analysis (Fig. 4). Furthermore, DCA results of predicting csPCa by radiomics models, PI-RADS assessed by three different level radiologists, and adjusted PI-RADS of three different level radiologists are summarized in Fig. 5.

**Fig. 6:**
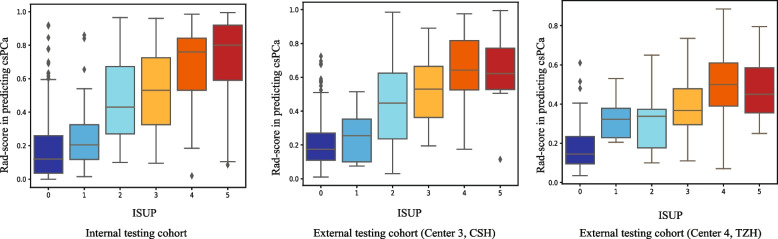
Box plots show the relationship between the Rad-score in predicting csPCa and ISUP in the internal testing cohort, an external testing cohort of center 3, and an external testing cohort of center 4. The participant-level correlation showed that the Rad-scores in predicting csPCa were positively correlated with ISUP in the internal testing cohort and external testing cohorts of center 3 and center 4. Notes: ISUP, International Society of Urological Pathology; center 3, CSH, Changshu NO.1 People’s Hospital; center 4, TZH, People’s Hospital of Taizhou; PI-RADS, Prostate Imaging Reporting and Data System

The relationship between Rad-scores produced by csPCa prediction models and ISUP was examined. The participant-level correlation showed that the Rad-score of the csPCa model was positively correlated with ISUP in the internal testing cohort (*r* = 0.690, *p* < 0.0001), the external testing cohort of center 3 (*r* = 0.700, *p* < 0.0001), and external testing cohort of center 4 (*r* = 0.688, *p* < 0.0001) (Fig. 6).

In the following steps, we further assessed the contribution of Radiomics Model in Predicting csPCa in Reducing the Proportion of Equivocal PI-RADS Category 3 Patients. PI-RADS lesions scored 3 are intermediate or equivocal lesions that pose a significant challenge to clinical management [6]. In this study, we found that the proportion of PI-RADS category 3 patients decreased in all three testing cohorts and overall participants (the overall decreased the percentage were 7.5%, 16.1%, and 2.1% of three different radiologists) (Figure S4) when assessed by radiologists with different levels of expertise who used radiomics model in predicting csPCa. The detailed proportion of increase and decrease of PI-RADS patients testing cohorts are summarized in Supplement Section 6.

## Discussion

Establishing a non-invasive precise diagnosis of csPCa and characterizing its pathologic properties are very important for predicting clinical outcomes and guiding the management of prostate disease [21]. In this retrospective, multi-center study, we developed and validated the radiomics model using four different algorithms to preoperatively predict csPCa and aggressiveness compared with PI-RADS obtained by radiologists with different experience levels. Our results obtained from a cohort of 1616 patients from 4 tertiary care medical centers showed that this radiomics model might accurately predict csPCa and aggressiveness and further help radiologists, especially junior doctors with less practical experience, improve their clinical diagnosis performance.

There are several innovations compared with previous studies. First, in our study, we applied ComBat for feature harmonization to alleviate the difference between the distribution of features among different centers and improve the performance of the models [20]. Second, we compared the diagnostic performance of radiologists with different clinical experiences using multi-center data, finding that the diagnosis performance of the radiomics model using the RF algorithm was comparable to PI-RADS_expert_ and PI-RADS_senior_ and superior to PI-RADS_junior_. Third, after integrating the radiomics model into the PI-RADS, the performance of adjusted PI-RADS was improved, which implied that the radiomics model could be a potential radiological assessment tool for radiologists. Finally, the Rad-scores of csPCa prediction models were positively correlated with ISUP in three testing cohorts, indicating that the Rad-score based on radiomics features can be used to characterize prostate cancer aggressiveness.

Previous single-center studies have used radiomics in predicting PCa and csPCa using MRI. Gong et al. [22] indicated that radiomics could non-invasively identify high-grade PCa. Chidozie and colleagues [16] showed that quantitative grey-level co-occurrence matrix (GLCM) texture analyses of MRI may be used as a non-invasive imaging technique to predict clinically significant cancer. Furthermore, Qi et al. [23] proved that the radiomics model could predict PCa in men with 4–10 ng/mL PSA. Gugliandolo and his team [24] performed a prospective trial and found that MRI-based radiomics is a promising tool for predicting PCa characteristics. Entirely consistent with the above research, our study further compared the diagnostic performance of radiomics models and radiologists and evaluated the role of radiomics in characterizing prostate cancer aggressiveness by predicting ISUP using multi-center data. It was found that this radiomics model could discriminate csPCa and even indicate the ISUP to characterize the aggressiveness of prostate cancer. In addition, in order to evaluate the generalization performance of the models, two independent external testing cohorts were used to test the ability and accuracy of the model in predicting csPCa. We found that the models achieved satisfactory predictions in both testing cohorts, implying the admirable generalization and stability of the radiomics models. To the best of our knowledge, this is the first study that addressed the generalizability of the radiomics models in the context of the classification of csPCa and ISUP based on multi-centric data from multiple vendors.

When the PI-RADS of radiologists were adjusted according to the radiomics predictions, the specificity was substantially increased while sensitivity was slightly decreased. The high sensitivity of PI-RADS by radiomics may lead to overdiagnosis and overtreatment in clinical practice [25]. On the other hand, the increase in specificity means that more patients could avoid immediate biopsy or RP [26]. The PI-RADS adjusted by the radiomics model may provide a more all-around tool to recommend surveillance for patients who might not require an instant treatment and maintain a comparatively high sensitivity for patients with aggressive prostate cancer. As revealed by DCA, the adjusted PI-RADS of different level radiologists showed greater net benefit than that based on PI-RADS assessment, which is to say the adjusting strategy by radiomics models can bring clinical benefits.

In clinical practice, the risk–benefit ratio of biopsy for PI-RADS category patients is still controversial. Taking PI-RADS_expert_ as an example, even though the PI-RADS was assessed by an expert radiologist with rich diagnostic experience in prostatic MRI, there were still 11.5% of patients with equivocal findings of csPCa, all of whom underwent painful biopsy, not to mention the PI-RADS assessed by junior or senior radiologists who had less experience in the diagnosis of prostate MRI. However, when the radiomics models were applied, the proportion of equivocal patients decreased to 9.4%, which implied that more patients could avoid unnecessary painful biopsies. Thus, the radiomics model can be used as an alternative way to predict csPCa in personalized medicine, especially with demanding clinical tasks and a shortage of expert-level radiologists.

The present study also has some limitations. First, not all patients underwent RP treatment for different clinical reasons; for some patients, biopsy pathology was used as a standard reference. In fact, some studies have reported that biopsy is a reliable way to detect PCa [27, 28]. Second, the validation of the model should be performed by future prospective multicenter studies. Third, although the diagnostic performance of the adjusted PI-RADS was improved in three testing cohorts, it is difficult to observe all statistically significant improvements in the performances given by the integration of the PI-RADS and radiomics model, probably due to the inconsistency of the multicenter dataset.

## Conclusion

In this study, we evaluated the generalizability of radiomics models in predicting csPCa with a large inhomogeneous cohort from four centers. This radiomics model is a powerful, non-invasive auxiliary tool for predicting csPCa aggressiveness, reducing unnecessary biopsies, and improving the diagnostic performance of PI-RADS of radiologists with different clinical experience.

### Supplementary Information

**Additional file 1: Supplement materials section 1.** MRI acquisition and PI-RADS assessment. **Supplement materials section 2.** MRI acquisition and PI-RADS assessment. **Supplement materials section 3.** Histopathology. **Supplement materials section 4.** **Supplement materials section 5.** **Supplement materials section 6.** Results. **Fig.** **S1.** The detailed pipeline of the machine learning models. Notes: Center 1, SUH1st, the first affiliated hospital of Soochow University; center 2, SUH2nd, the second affiliated hospital of Soochow University; center 3, CSH, Changshu NO.1 People’s Hospital; center 4, TZH, People’s Hospital of Taizhou; PCa, Prostate Cancer. ML, machine learning. **Fig. S2.** The diagnosis performance of the PI-RADS of three different radiologists in predicting csPCa in the internal testing cohort and external testing cohorts of center 3 and center 4. ROC, receiver operating characteristics; AUC, area under ROC curve; ACC, accuracy; SEN, sensitivity; SPE, specificity; center 3, CSH, Changshu NO.1 People’s Hospital; center 4, TZH, People’s Hospital of Taizhou; csPCa: Clinically Significant Prostate Cancer; PI-RADS: Prostate Imaging Reporting and Data System. **Fig. S3.** The comparison of the diagnosis performance of radiomics models using four different machine learning algorithms in predicting csPCa in the internal testing cohort, an external testing cohort of center 3, and an external testing cohort of center 4. The radiomics model using RF algorithm had the highest predictive performance in all external testing cohorts; the differences were statistically significant in the internal testing cohort and an external testing cohort of center 3; however, the difference was insignificant in the external testing cohort of center 4. Notes: ROC, receiver operating characteristics; AUC, area under ROC curve; ACC, accuracy; SEN, sensitivity; SPE, specificity; center 3, CSH, Changshu NO.1 People’s Hospital; center 4, TZH, People’s Hospital of Taizhou; RF: random forest, SVM: support vector machine, LR: logistic regression and LDA: Linear Discriminant Analysis; csPCa: Clinically Significant Prostate Cancer. **Fig.** **S4.** The total proportion of PI-RADS category 3 patients of junior, senior, and expert radiologists in all testing cohorts when the radiomics model in predicting csPCa was used to adjust the PI-RADS. The proportion of equivocal findings decreased to various degrees. **Table S1.** Parameters of MRI scanning from four institutions. **Table S2.** Selected features in predicting csPCa. **Table S3.** The Cross-validation results of Radiomics models using four different machine learning algorithms in predicting csPCa in training cohort.

## Data Availability

The imaging studies and clinical data used for algorithm development are not publicly available, because they contain private patient health information. Interested users may request access to these data, where institutional approvals along with signed data use agreements and/or material transfer agreements may be needed/negotiated. Derived result data supporting the findings of this study are available upon reasonable requests.

## Abbreviations

ADC: Apparent diffusion coefficient
ciPCa: Clinically insignificant prostate cancer
csPCa: Clinically significant prostate cancer
DCA: Decision curve analysis
DCE: Dynamic contrast enhanced
DWI: Diffusion weighted imaging
ISUP: International Society of Urological Pathology
LDA: Linear discriminant analysis
LR: Logistic regression
mpMRI: Multiparametric magnetic resonance imaging
PCa: Prostate cancer
PCC: Pearson correlation coefficient
PI-RADS: Prostate Imaging Reporting and Data System
PSA: Prostate specific antigen
Rad-score: Radiomics score
RF: Random forest
ROC: Receiver operating characteristic
RP: Radical prostatectomy
SEN: Sensitivity
SPE: Specificity
SVM: Support vector machine
T2WI: T2 weighted imaging
TRUS: Transrectal ultrasound
