# Supplementary material for: Prediction of clinically significant prostate cancer using radiomics models in real-world clinical practice: a retrospective multicenter study
Source: Insights Imaging. 2024 Feb 29;15:68. doi: 10.1186/s13244-024-01631-w (PMC10904705; doi:10.1186/s13244-024-01631-w)
Supplement: Supplementary file 1 — Additional file 1: Supplement materials section 1. MRI acquisition and PI-RADS assessment. Supplement materials section 2. MRI acquisition and PI-RADS assessment. Supplement materials section 3. Histopathology. Supplement materials section 4. Supplement materials section 5. Supplement materials section 6. Results. Fig. S1. The detailed pipeline of the machine learning models. Notes: Center 1, SUH1st, the first affiliated hospital of Soochow University; center 2, SUH2nd, the second affiliated hospital of Soochow University; center 3, CSH, Changshu NO.1 People’s Hospital; center 4, TZH, People’s Hospital of Taizhou; PCa, Prostate Cancer. ML, machine learning. Fig. S2. The diagnosis performance of the PI-RADS of three different radiologists in predicting csPCa in the internal testing cohort and external testing cohorts of center 3 and center 4. ROC, receiver operating characteristics; AUC, area under ROC curve; ACC, accuracy; SEN, sensitivity; SPE, specificity; center 3, CSH, Changshu NO.1 People’s Hospital; center 4, TZH, People’s Hospital of Taizhou; csPCa: Clinically Significant Prostate Cancer; PI-RADS: Prostate Imaging Reporting and Data System. Fig. S3. The comparison of the diagnosis performance of radiomics models using four different machine learning algorithms in predicting csPCa in the internal testing cohort, an external testing cohort of center 3, and an external testing cohort of center 4. The radiomics model using RF algorithm had the highest predictive performance in all external testing cohorts; the differences were statistically significant in the internal testing cohort and an external testing cohort of center 3; however, the difference was insignificant in the external testing cohort of center 4. Notes: ROC, receiver operating characteristics; AUC, area under ROC curve; ACC, accuracy; SEN, sensitivity; SPE, specificity; center 3, CSH, Changshu NO.1 People’s Hospital; center 4, TZH, People’s Hospital of Taizhou; RF: random forest, SVM: support vector [file 13244_2024_1631_MOESM1_ESM.pdf]

# Prediction of Clinically Significant Prostate Cancer using Radiomics Models in Real-World Clinical Practice: A Retrospective Multicenter Study

## ELECTRONIC SUPPLEMENTARY MATERIAL

## Supplement materials section 1

## MRI acquisition and PI-RADS assessment

Second, after at least 4 weeks, PI-RADS assessment of the two board-certified radiologists, namely PI-$RADS_{senior}$, were re-scored by two senior-level radiologists from center 1 and center 4 (reader 3 and 4 with 9 and 10 years of experience in prostate imaging, respectively). Third, after at least 4 weeks, two expert-level board-certified radiologists from center 1 (reader 5 and 6 with 24 and 20 years of experience in prostate imaging, respectively) accessed PI-RADS for the third time, namely PI-$RADS_{expert}$, of all the patients. All the readers were blind to the other reader's scores in the process of assessment. As for the csPCa prediction, we set the cut-off value at PI-RADS ≥3. All investigators had access to the clinical details, including age, PSA level, digital rectal examination, and other risk factors (like family history and routine habits). All readers were members of the institution's prostate disease management team with 3-24 years' experience in prostatic MRI (they had read >1000 prostatic MRI scans using a PI-RADS scoring system before the present study). All readers were blinded to histopathologic data. As it is challenging to achieve a per-lesion imaging correlation with prostatectomy specimens in retrospective data, the unit of assessment in this study was per-patient. If patients had multiple lesions, only the index lesion with the highest Gleason score or the largest size (if it had the same Gleason score) was assessed.

**Supplement materials section 2**

**MRI acquisition and PI-RADS assessment**

Manual segmentations were performed by reader 1 and reader 3. The main challenge in imaging labeling was the presence of ambiguous regions where the tumor boundary could hardly be deduced from the image. Thus, the contours of VOIs were re-checked in consensus with the above two expert radiologists (reader 5 and reader 6). All the radiologists in the segmentation of the VOIs were blinded to the PI-RADS, clinical data, and histopathologic results.

**Supplement materials section 3**

**Histopathology**

Patients who scored PI-RADS 1-2 without suspicious lesions underwent TRUS-guided systemic biopsy. Patients with PCa confirmed by biopsy underwent radical prostatectomy (RP), active surveillance (AS) or androgen deprivation therapy (ADT), or radiation therapy (RT).

For patients who underwent both biopsy and RP, the pathology of RP was used for the final result. Histopathology findings were recorded using the following criteria: benign (ISUP grade 0, Gleason score, GS<3+3), ISUP grade 1 (GS:3+3), ISUP grade 2 (GS:3+4), ISUP grade 3 (GS:4+3), ISUP grade 4 (GS: 8), and ISUP grade 5 (GS:>8). According to the ISUP grading system [1-3], ISUP≤ 2 as non-clinically significant prostate cancer (non-csPCa), and ISUP>2 was defined as csPCa. In the present study, ISUP ≤ 2 was nominated as the non-csPCa group, and ISUP>2 as csPCa group. As for patients who only underwent biopsy without RP, their pathology results of the biopsy were used.

**Supplement materials section 4**

We extracted radiomics features of VOIs from T2WI, DWI, and ADC sequences, fully exploiting relevant features and providing complete information about the tumor, including shape, intensity, intratumor heterogeneity, etc. According to the image biomarker standardization initiative (IBSI) suggestion, we first normalized the T2WI and DWI of each case to the range of 1-1000, and then the ADC maps based on the whole training cohort. For the sequence registration, we used Elastix (v.5.0.1) to align DWI and ADC on the T2W images. Next, we resampled the image to an intra-slice resolution of 0.5 mm x 0.5 mm by spline. Morphology features were extracted on the original ROIs, and intensity-based features were extracted with 3-sigma re-segmentation. Consequently, we discretized the tumor of all three sequences to 16 bins by analyzing the whole cohort to extract texture features, including 51 first-order features, 14 shape-based features, 72 features of grey-level co-occurrence matrix (GLCM), 48 of grey level run length matrix (GLRLM), 48 of grey level size zone matrix (GLSZM), 44 of grey level distance zone matrix (GLDZM), and 15 of neighborhood grey tone difference matrix (NGTDM). All texture features were calculated by a 2.5D strategy, which means that the transform matrix and the features were estimated by in-plane, after which the final features were merged across all planes. Two senior radiologists randomly selected 30 cases to re-delineate the lesions and calculate the between-group correlation coefficients. Also, the consistency between the two radiologists in delineating VOIs was 0.81. The details of pipeline of the model were declared in **Fig.S1**.

## Supplement materials section 5

Image protocol quality - well-documented image protocols (for example, contrast, slice thickness, energy, etc.) and/or usage of public image protocols allow reproducibility/replicability

- [x] protocols well documented
- [ ] public protocol used
- [ ] none

Multiple segmentations - possible actions are: segmentation by different physicians/algorithms/software, perturbing segmentations by (random) noise, segmentation at different breathing cycles. Analyse feature robustness to segmentation variabilities

- (●) yes
- ( ) no

Phantom study on all scanners - detect inter-scanner differences and vendor-dependent features. Analyse feature robustness to these sources of variability

- ( ) yes
- (●) no

Imaging at multiple time points - collect images of individuals at additional time points. Analyse feature robustness to temporal variabilities (for example, organ movement, organ expansion/shrinkage)

- ( ) yes
- (●) no

Feature reduction or adjustment for multiple testing - decreases the risk of overfitting. Overfitting is inevitable if the number of features exceeds the number of samples. Consider feature robustness when selecting features

- (●) Either measure is implemented
- ( ) Neither measure is implemented

Multivariable analysis with non radiomics features (for example, EGFR mutation) - is expected to provide a more holistic model. Permits correlating/inferencing between radiomics and non radiomics features

- (●) yes
- ( ) no

Detect and discuss biological correlates - demonstration of phenotypic differences (possibly associated with underlying gene–protein expression patterns) deepens understanding of radiomics and biology

- ( ) yes
- (●) no

Cut-off analyses - determine risk groups by either the median, a previously published cut-off or report a continuous risk variable. Reduces the risk of reporting overly optimistic results

- ( ) yes

◉ no

Discrimination statistics - report discrimination statistics (for example, C-statistic, ROC curve, AUC) and their statistical significance (for example, p-values, confidence intervals). One can also apply resampling method (for example, bootstrapping, cross-validation)

☑ a discrimination statistic and its statistical significance are reported

☑ a resampling method technique is also applied

☐ none

Calibration statistics - report calibration statistics (for example, Calibration-in-the-large/slope, calibration plots) and their statistical significance (for example, P-values, confidence intervals). One can also apply resampling method (for example, bootstrapping, cross-validation)

☑ a calibration statistic and its statistical significance are reported

☑ a resampling method technique is applied

☐ none

Prospective study registered in a trial database - provides the highest level of evidence supporting the clinical validity and usefulness of the radiomics biomarker

○ yes

◉ no

Validation - the validation is performed without retraining and without adaptation of the cut-off value, provides crucial information with regard to credible clinical performance

☐ No validation

☐ validation is based on a dataset from the same institute

☐ validation is based on a dataset from another institute

☐ validation is based on two datasets from two distinct institutes

☐ the study validates a previously published signature

☑ validation is based on three or more datasets from distinct institutes

Comparison to 'gold standard' - assess the extent to which the model agrees with/is superior to the current 'gold standard' method (for example, TNM-staging for survival prediction). This comparison shows the added value of radiomics

◉ yes

○ no

Potential clinical utility - report on the current and potential application of the model in a clinical setting (for example, decision curve analysis).

◉ yes

○ no

Cost-effectiveness analysis - report on the cost-effectiveness of the clinical application (for example, QALYs generated)

○ yes

◉ no

Open science and data - make code and data publicly available. Open science facilitates knowledge transfer and reproducibility of the study

☐ scans are open source

☐ region of interest segmentations are open source

☑ the code is open sourced

☐ radiomics features are calculated on a set of representative ROIs and the calculated features and representative ROIs are open source

Total score

20
(55.56%)

**Supplement materials section 6**

**Results**

In the internal testing cohort, the proportion of PI-RADS equivocal 3 patients decreased from 22.3% to 17.7% when assessed by junior radiologists, from 22.6% to 13.8% when assessed by senior radiologists and from 17.4% to 13.8% when assessed by expert radiologists, respectively. In an external testing cohort of Center 3, the proportion of PI-RADS category 3 patients decreased from 28% to 25.4% when assessed by junior radiologists, and from 34.8% to 7.2% when assessed by senior radiologists. As to the PI-RADS assessed by expert radiologists, the proportion increased from 3.9% to 9.7%, of which the proportion is still relatively low. In an external testing cohort of Center 4, the proportion of PI-RADS category 3 patients decreased from 25.8% to 8.9% when assessed by junior radiologists, and from 21% to 8.1% when assessed by senior radiologists, from 12.1% to 3.1% when assessed by expert radiologists.

As for total proportion of PI-RADS category 3 patients in all testing cohorts, as shown in Figure S4, the proportion of PI-RADS category 3 patients decreased from 25.2% to 24.6% when assessed by junior radiologists, from 26.1% to 10% when

assessed by senior radiologists, and from 11.5% to 9.4% when assessed by expert radiologists. In the worst-case scenario, all patients with equivocal findings would undergo invasive prostatic biopsy. When the radiomics models were used to adjust the PI-RADS of different radiologists, the proportion of patients who underwent biopsy decreased by varying degrees when assessed by of all radiologists.

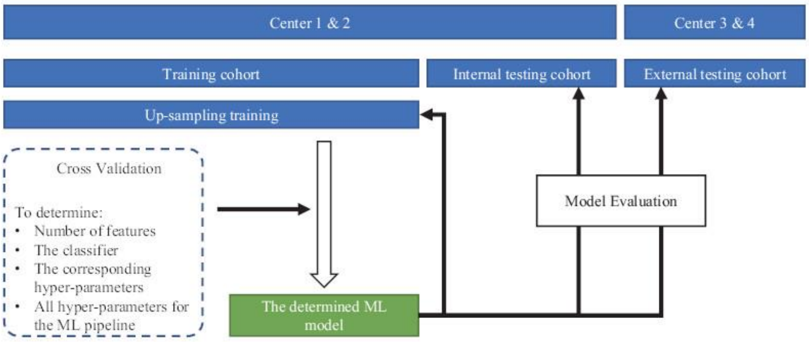


**Fig.S1. The detailed pipeline of the machine learning models.** Notes: Center 1, SUH1st, the first affiliated hospital of Soochow University; Center 2, SUH2nd, the second affiliated hospital of Soochow University; Center 3, CSH, Changshu NO.1 People's Hospital; Center 4, TZH, People's Hospital of Taizhou; PCa, Prostate Cancer. ML, machine learning.

Diagnosis performance of PI-RADS of three radiologists in internal and external testing cohorts for csPCa

| PI-RADS | Internal testing cohort (n=327) | | | | | External testing cohort (CSH) n = 279 | | | | | External testing cohort (TZH) n = 248 | | | | |
|---|---|---|---|---|---|---|---|---|---|---|---|---|---|---|---|
| ≥3 | AUC(95%CI) | SEN | SPE | PPV | NPV | AUC | SEN | SPE | PPV | NPV | AUC | SEN | SPE | PPV | NPV |
| PI-RADSjunior | 0.845 (0.796-0.894) | 91.3 (95/104) | 44.4 (99/223) | 43.4 (95/219) | 91.7 (99/108) | 0.823 (0.765-0.882) | 95.4 (62/65) | 22.0 (47/214) | 27.1 (62/229) | 94.0 (47/50) | 0.858 (0.808-0.908) | 94.2 (113/120) | 45.3 (58/128) | 61.7 (113/183) | 89.2 (58/65) |
| PI-RADSsenior | 0.877 (0.836-0.919) | 95.2 (99/104) | 48.9 (109/223) | 46.5 (99/213) | 95.6 (109/114) | 0.856 (0.807-0.904) | 98.5 (64/65) | 45.3 (97/214) | 35.4 (64/181) | 99.0 (97/98) | 0.867 (0.818-0.916) | 94.2 (113/120) | 56.3 (72/128) | 66.9 (113/169) | 91.1 (72/79) |
| PI-RADSexpert | 0.892 (0.855-0.929) | 97.1 (101/104) | 52.5 (117/223) | 48.8 (101/207) | 97.5 (117/120) | 0.884 (0.838-0.930) | 93.8 (61/65) | 75.2 (161/214) | 53.5 (61/114) | 97.6 (161/165) | 0.888 (0.843-0.932) | 92.5 (111/120) | 75.0 (96/128) | 77.6 (111/143) | 91.4 (96/105) |

Note: AUC=area under the receiver operating characteristic curve; SEN=sensitivity; ACC= Accuracy; SEN=sensitivity; SPE: specificity; PPV, positive predictive value; NPV, negative predictive value;
csPCa= clinically significant prostate cancer

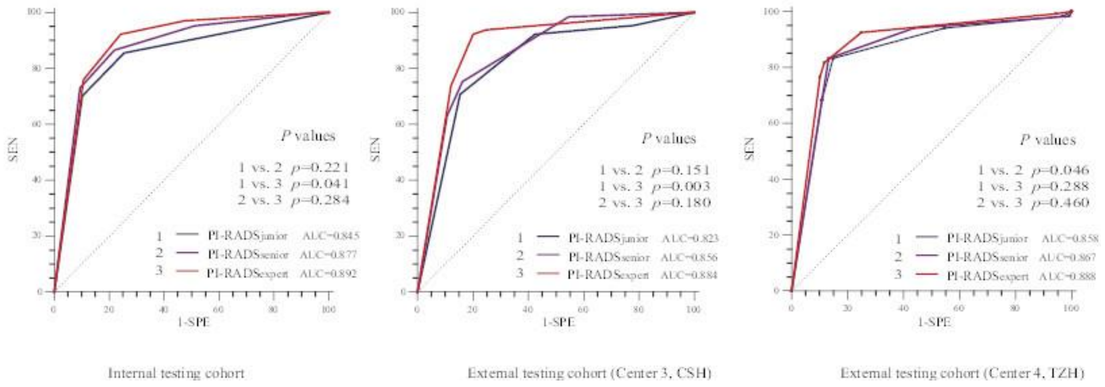


**Fig. S2. The diagnosis performance of the PI-RADS of three different radiologists in predicting csPCa in the internal testing cohort and external testing cohorts of Center 3 and Center 4.** ROC, receiver operating characteristics; AUC, area under ROC curve; ACC, accuracy; SEN, sensitivity; SPE, specificity; Center 3, CSH, Changshu NO.1 People's Hospital; Center 4, TZH, People's Hospital of Taizhou; csPCa: Clinically Significant Prostate Cancer; PI-RADS: Prostate Imaging Reporting and Data System.

Diagnosis performance of Radiomics model with different machine learning algorithms in testing cohorts for csPCa

| | Internal testing cohort | | | | | External testing cohort (CSH) | | | | | External testing cohort (TZH) | | | | |
|---|---|---|---|---|---|---|---|---|---|---|---|---|---|---|---|
| | AUC(95%CI) | SEN | SPE | PPV | NPV | AUC(95%CI) | SEN | SPE | PPV | NPV | AUC | SEN | SPE | PPV | NPV |
| RF | 0.874(0.834-0.915) | 83.7 (87/104) | 78 (174/223) | 64.0 (87/136) | 91.1 (174/191) | 0.876(0.831-0.920) | 87.7 (57/65) | 77.6 (166/214) | 54.3 (57/105) | 95.4(166/174) | 0.893(0.853-0.933) | 90.0 (108/120) | 73.2 (94/128) | 76.6 (108/142) | 88.8 (94/106) |
| SVM | 0.853(0.808-0.899) | 80.8 (84/104) | 81.2 (181/223) | 66.7 (84/126) | 90.1 (181/201) | 0.831(0.772-0.891) | 75.4 (49/65) | 82.7 (177/214) | 57 (49/86) | 91.7(177/193) | 0.858(0.807-0.909) | 86.7 (104/120) | 80.5 (103/128) | 80.6 (104/129) | 86.7 (103/119) |
| LR | 0.852(0.807-0.897) | 87.5 (91/104) | 73.5 (164/223) | 60.7 (91/150) | 92.7 (164/177) | 0.839(0.781-0.898) | 72.3 (47/65) | 86.5 (185/124) | 61.8 (47/76) | 91.1 (185/203) | 0.878(0.833-0.924) | 84.2(101/120) | 85.2(109/128) | 84.2(101/120) | 85.2(109/128) |
| LDA | 0.714(0.661-0.768) | 63.5 (66/104) | 79.4 (177/223) | 58.9 (66/112) | 82.3 (177/215) | 0.655(0.588-0.723) | 55.4 (36/65) | 75.7 (162/214) | 40.9 (36/88) | 84.8(162/191) | 0.646(0.593-0.698) | 40.8 (49/120) | 88.3 (113/128) | 76.6 (49/64) | 61.4 (113/184) |

Note: AUC=area under the receiver operating characteristic curve; SEN=sensitivity; ACC= Accuracy; SEN=sensitivity; SPE: specificity; PCa= prostate cancer; CSPC= clinically significant prostate cancer; SVM. RF:random forest; LR: Logistic Regression; DT: Decision Tree

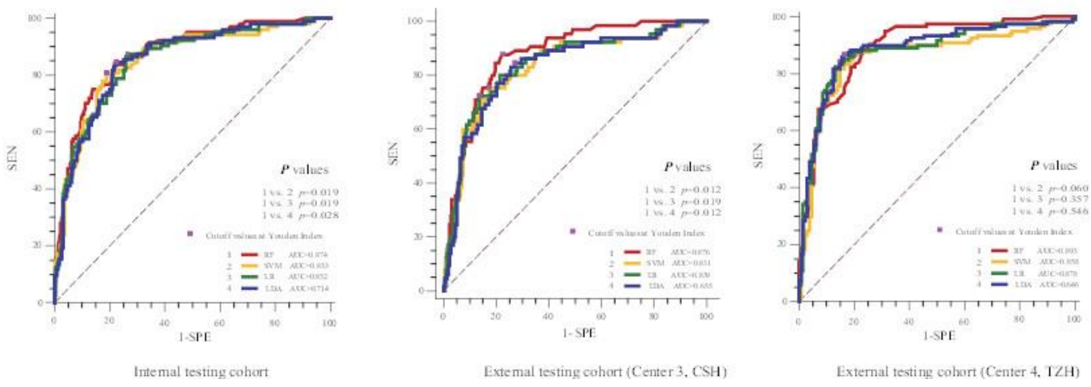


**Fig. S3. The comparison of the diagnosis performance of radiomics models using four different machine learning algorithms in predicting csPCa in the internal testing cohort, an external testing cohort of Center 3, and an external testing cohort of Center 4.** The radiomics model using RF algorithm had the highest predictive performance in all external testing cohorts; the differences were statistically significant in the internal testing cohort and an external testing cohort of Center 3; however, the difference was insignificant in the external testing cohort of Center 4. Notes: ROC, receiver operating characteristics; AUC, area under ROC curve; ACC, accuracy; SEN, sensitivity; SPE, specificity; Center 3, CSH, Changshu NO.1 People's Hospital; Center 4, TZH, People’s Hospital of Taizhou; RF: random forest, SVM: support vector machine, LR: logistic regression and LDA: Linear Discriminant Analysis; csPCa: Clinically Significant Prostate Cancer.

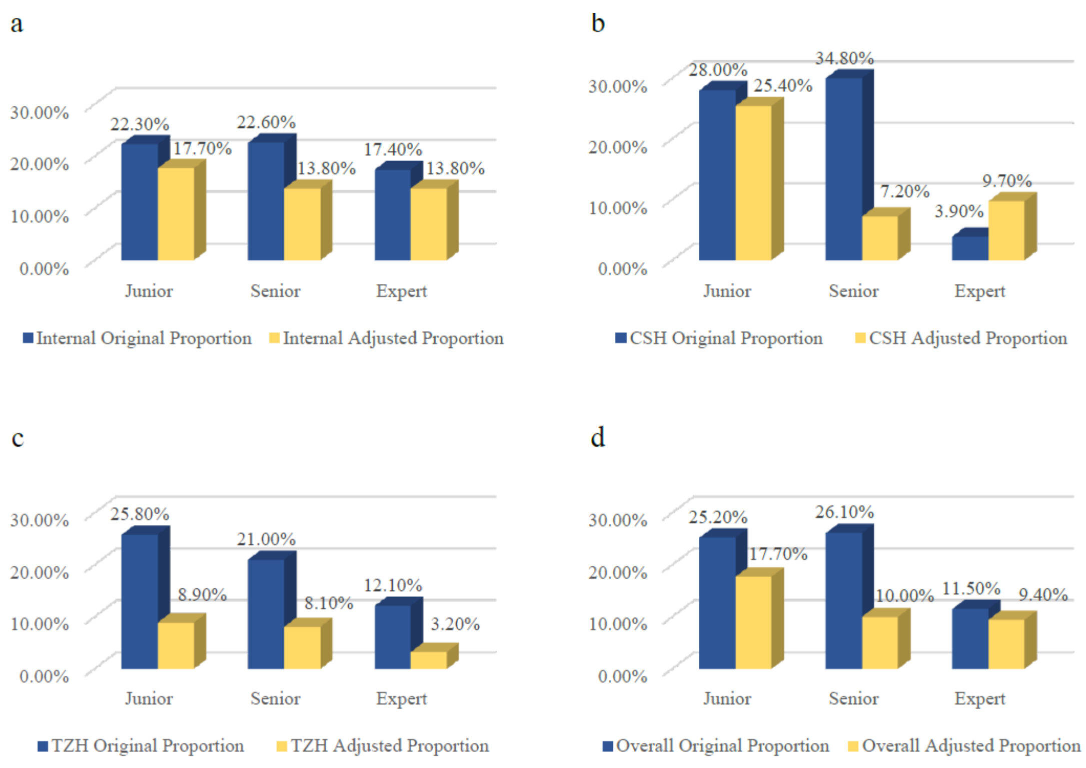


**Fig.S4.** The total proportion of PI-RADS category 3 patients of junior, senior, and expert radiologists in all testing cohorts when the radiomics model in predicting csPCa was used to adjust the PI-RADS. The proportion of equivocal findings decreased to various degrees.

**Table S1.** Parameters of MRI scanning from four institutions

| Center | Sequences | Vendor | MRI strength | B value(sec/mm$^2$) | Slice thickness (mm) | Spacing between slices (mm) | Echo time(s) | Repetition time(s) |
|---|---|---|---|---|---|---|---|---|
| SUH 1st | T2WI, DWI, ADC | Siemens Skyra | 3.0 T | 50/70/1500/2000 | 3 | 3/3.45 | 60/104 | 6540/7590 |
| SUH 2nd | T2WI, DWI, ADC | Philips Ingenia | 3.0 T | 10/20/50/100/200/1000/2000 | 1.5/2/3/3.4/3.5/3.7/3.8/3.9/4/4.1/4.2/4.3/5 | 1.65/3/3.2/3.3/3.4/3.5/3.7/3.8/3.9/2.2/4/4.1/4.2/4.3/5 | 77/78/100 | 4542/4828/4898/4733/4972/ 6000 |
| TZH | T2WI, DWI, ADC | Siemens Skyra and Vero | 3.0 T | 0/50/800/1000/1500 | 3.5/4/5/5.5 | 3.5/4/4.8/6/6.6 | 62/64/74/97/104 | 4480/5000/5100/7500/8600 |
| CSH | T2WI, DWI, ADC | Philips Achieva TX | 3.0 T | 0, 1000, 2000 | 3 | 3 | 76/80 | 2750/3000 |

*Notes: T2WI, T2-weighted imaging; DWI, diffusion-weighted imaging; ADC, Apparent diffusion coefficient; SUH 1st, the First Affiliated Hospital of Soochow University; SUH 2nd, the Second Affiliated Hospital of Soochow University; CSH, Changshu NO.1 People's Hospital; TZH, the People's Hospital of Taizhou*

**Table S2**. Selected features in predicting csPCa

| |
|---|
| original_shape_Sphericity |
| T2WI_original_firstorder_90Percentile |
| T2WI_original_firstorder_Mean |
| T2WI_original_firstorder_RootMeanSquared |
| T2WI_original_glcm_Imc1 |
| T2WI_original_glcm_Imc2 |
| T2WI_original_glszm_SmallAreaEmphasis |
| T2WI_original_glszm_ZoneEntropy |
| DWI_original_firstorder_90Percentile |
| DWI_original_firstorder_Maximum |
| DWI_original_firstorder_MeanAbsoluteDeviation |
| DWI_original_firstorder_Range |
| DWI_original_glcm_InverseVariance |
| DWI_original_gldm_DependenceNonUniformityNormalized |
| DWI_original_gldm_DependenceVariance |
| DWI_original_gldm_LargeDependenceHighGrayLevelEmphasis |
| DWI_original_glszm_GrayLevelVariance |
| DWI_original_glszm_ZoneEntropy |
| ADC_original_firstorder_10Percentile |
| ADC_original_firstorder_90Percentile |
| ADC_original_firstorder_Maximum |
| ADC_original_firstorder_Median |
| ADC_original_firstorder_Minimum |
| ADC_original_firstorder_RootMeanSquared |
| ADC_original_glcm_MCC |

**Table S3.** The Cross-validation results of Radiomics models using four different machine learning algorithms in predicting csPCa in training cohort

| Algorithms | AUC (95%CI) | SEN | SPE | PPV | NPV |
|---|---|---|---|---|---|
| RF | 0.816 (0.784-0.849) | 70.8(172/243) | 79.6(413/519) | 61.9(172/278) | 85.3(413/484) |
| SVM | 0.804 (0.770-0.838) | 63.8(155/243) | 82.9(430/519) | 63.5(155/244) | 83.0(430/518) |
| LR | 0.810(0.777-0.844) | 60.5(147/243) | 86.7(450/519) | 68.1(147/216) | 82.4(450/546) |
| LDA | 0.793(0.759-0.828) | 62.6(152/243) | 81.3(422/519) | 61.0(152/249) | 82.3(422/513) |

*Notes: RF, the random forest; SVM, support vector machine; LR, logistic regression; LDA, Linear Discriminant Analysis; SEN, sensitivity; SPE, specificity; PPV, positive predict value; NPV, negative predict value; ROC, receiver operating characteristic curves; AUC, the area under the ROC curve; csPCa, clinically significant prostate cancer*

## References

[1] Abdollah F, Sun M, Schmitges J, Tian Z, Jeldres C, Briganti A, Shariat SF, Perrotte P, Montorsi F, Karakiewicz PI. Cancer-specific and other-cause mortality after radical prostatectomy versus observation in patients with prostate cancer: competing-risks analysis of a large North American population-based cohort. Eur Urol. 2011;60:920-30.

[2] Ahmed HU, El-Shater Bosaily A, Brown LC, Gabe R, Kaplan R, Parmar MK, Collaco-Moraes Y, Ward K, Hindley RG, Freeman A, Kirkham AP, Oldroyd R, Parker C, Emberton M. Diagnostic accuracy of multi-parametric MRI and TRUS biopsy in prostate cancer (PROMIS): a paired validating confirmatory study. The Lancet. 2017;389:815-22.

[3] Ahdoot M, Wilbur AR, Reese SE, Lebastchi AH, Mehralivand S, Gomella PT, Bloom J, Gurram S, Siddiqui M, Pinsky P, Parnes H, Linehan WM, Merino M, Choyke PL, Shih JH, Turkbey B, Wood BJ, Pinto PA. MRI-Targeted, Systematic, and Combined Biopsy for Prostate Cancer Diagnosis. N Engl J Med. 2020;382:917-28.
